# Coronary Neointimal Healing After Intravascular Lithotripsy Compared With Orbital Atherectomy Assessed by Serial Optical Coherence Tomography

**DOI:** 10.1016/j.jscai.2024.101977

**Published:** 2024-04-16

**Authors:** Anna Tsioulias, Doosup Shin, Mandeep Singh, Susan V. Thomas, Koshiro Sakai, Akiko Maehara, Omar Khalique, Evan S. Shlofmitz, Jeffrey W. Moses, Allen Jeremias, Richard A. Shlofmitz, Ziad A. Ali

**Affiliations:** a Department of Cardiology, St Francis Hospital, Roslyn, New York; b Cardiovascular Research Foundation, New York, New York; c Department of Medicine, Division of Cardiology, Columbia University Irving Medical Center, New York, New York; d New York Institute of Technology, Old Westbury, New York

**Keywords:** intravascular lithotripsy, neointimal healing, optical coherence tomography, orbital atherectomy

Coronary atherectomy has been used to modify heavily calcified coronary lesions prior to implantation of drug-eluting stents (DES) during percutaneous coronary intervention (PCI). Intravascular lithotripsy (IVL) has emerged as a novel therapy for the treatment of coronary calcification, which is increasingly used in clinical practice due to its safety, effectiveness, and ease of use.^1^^,^^2^ Recently, we first described optical coherence tomography (OCT) visualization of the healing process after IVL-assisted PCI with DES implantation.^3^ However, the effect of IVL on neointimal healing after implantation of DES in calcified coronary lesions, compared with that of atherectomy, has not been previously evaluated. Therefore, we sought to investigate the effects of orbital atherectomy (OA) and IVL on the neointimal healing process after DES implantation using serial OCT.

We retrospectively identified 19 patients (median age, 73 years; male, 57.9%) who underwent successful PCI with DES implantation after lesion preparation with OA (n = 8) or IVL (n = 11) and returned for clinically indicated follow-up catheterization at a median of 10 months IQR, 6-16) after the index PCI. There was no significant difference in baseline patient demographics and comorbidities between the OA and IVL groups. OCT was performed at baseline, after OA or IVL, after DES implantation, and during follow-up. Stent expansion was calculated by minimal stent area (MSA)/reference vessel lumen area by OCT. Cross-sectional areas of lumen, stent, intrastent lumen, and intrastent tissue were analyzed frame-by-frame at 0.2 mm intervals, which were then used to perform a volumetric analysis: volume or volumetric area (mm^3^) = ∑ cross-section area (mm^2^) × 0.2 mm. From follow-up OCT, neointimal volume (mm^3^) was calculated as volume of tissue growth between stent struts and intrastent lumen. Percent neointimal volume (%) was calculated as neointimal volume (mm^3^)/volumetric stent area (mm^3^). Volumetric measures were compared after being normalized to a 1 mm segment (mm^3^/mm; indexed volumetric area or indexed volume) to account for differences in stent length. All data are presented as median and IQR and compared by using the Mann-Whitney test. The study protocol was compliant with the Declaration of Helsinki and approved by the institutional review board at St. Francis Hospital. The need for informed consent for retrospective case review was waived due to minimal risk.

On pre-PCI OCT, OA and IVL groups showed comparable lesion characteristics including minimal lumen area (1.92 mm^2^ [1.06; 3.68] vs 1.63 mm^2^ [1.31; 2.11], respectively; *P* = .87), lesion length (30.2 ± 10.2 mm vs 25.7 ± 10.2 mm; *P* = .35), maximum calcium thickness (1.1 ± 0.2 mm vs 1.2 ± 0.2 mm; *P* = .24), arc (338.9° [275.0; 360.0] vs 360.0° [265.0; 360.0]; *P* = .75), and length (25.7 ± 10.4 mm vs 23.4 ± 9.6 mm; *P* = .62). All OA and IVL patients received second-generation DES with mean stent diameter of 3.2 ± 0.6 mm vs 3.3 ± 0.5 mm, respectively, and achieved excellent stent expansion (OA, 91.9% [83.1, 99.6]; IVL, 91.8% [89.4, 94.6]; *P* = .80). There were no significant differences in post-PCI OCT parameters between IVL and OA patients: MSA (OA, 5.2 mm^2^ [4.2, 7.2]; IVL, 5.2 mm^2^ [4.3, 6.2]; *P* = .80) and indexed volumetric stent area (OA, 7.3 mm^3^/mm [6.3, 9.8]; IVL, 7.4 mm^3^/mm [6.2, 8.3]; *P* = .80). Representative OCT images from each group are presented in Figure 1A and B, demonstrating interval coverage of stent struts by neointimal tissue. On follow-up OCT, the percentage of uncovered stent struts was low and comparable between the 2 groups (OA, 2.2% [1.2, 4.1] vs IVL, 3.0% [1.4, 6.2]; *P* = .72). Furthermore, there were no statistically significant differences between the OA and IVL groups with regards to MSA (OA, 3.60 ± 2.72; IVL, 3.66 ± 1.73; *P* = .95), the change in indexed volumetric lumen area of the stented segment (OA, –21.2 mm^3^ [–29.8, –11.5]; IVL, –16.3 mm^3^ [–20.9, –8.0]; *P* = .36), indexed neointimal volume (OA, 1.5 mm^3^/mm [1.2, 1.9]; IVL, 1.2 mm^3^/mm [0.6, 2.0]; *P* = .87), and percent neointimal volume (OA, 23.6% [15.8, 24.6]; IVL, 19.8% [11.0, 29.8]; *P* = .56) (Figure 1C).

**Figure 1:**
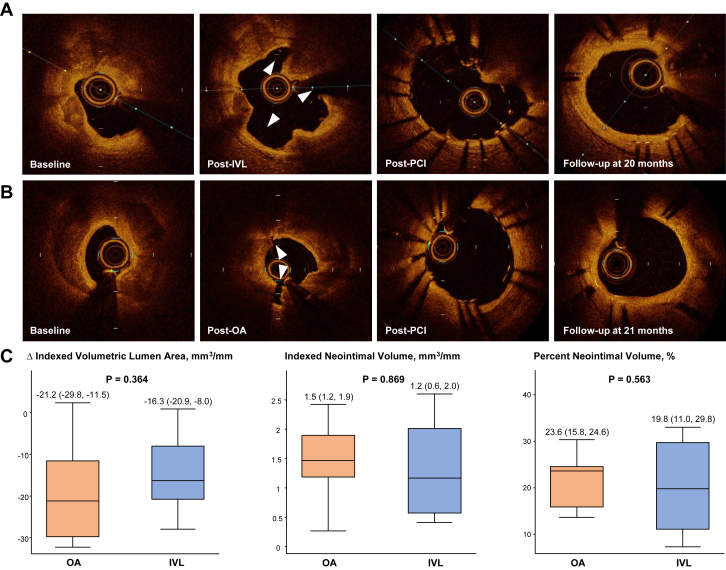
**Neointimal healing response assessed by optical coherence tomography (OCT) after orbital atherectomy (OA)- or intravascular lithotripsy (IVL)-assisted percutaneous coronary intervention (PCI) with drug-eluting stent (DES) implantation.** (**A** and **B**) Representative matched OCT cross-sections at baseline, after OA or IVL, after DES implantation, and during follow-up. White arrowheads indicate calcium fracture after OA or IVL. Follow-up OCT cross-sections demonstrate healing process and growth of neointimal tissue covering stent struts. (**C**) Quantitative parameters of neointimal healing process between OA- and IVL-assisted DES implantation are presented in box and whisker plots. Horizontal line indicates median value, box indicates the interquartile range, and whiskers indicate the minimum and maximum values excluding outliers.

The present study is the first comparative OCT analysis of neointimal healing response after OA- vs IVL-assisted DES implantation. The results of the present study suggest that neointimal healing response at a median follow-up of 10 months was not different between OA- and IVL-assisted PCI with DES implantation, supporting the use of either device for the treatment of heavily calcified coronary lesions to facilitate stent implantation. However, potential bias could not be excluded due to the small sample size, and various follow-up durations might have affected outcomes. Larger studies with clinical outcomes are warranted to investigate the long-term effects of OA and IVL-assisted DES implantation.

## References

[1] Ali Z.A., Nef H., Escaned J. (2019). Safety and effectiveness of coronary intravascular lithotripsy for treatment of severely calcified coronary stenoses: the disrupt CAD II study. Circ Cardiovasc Interv.

[2] Hill J.M., Kereiakes D.J., Shlofmitz R.A. (2020). Intravascular lithotripsy for treatment of severely calcified coronary artery disease. J Am Coll Cardiol.

[3] Chau K.W., Shlofmitz E.S., Jeremias A., Shlofmitz R.A., Ali Z.A. (2023). Optical coherence tomography assessed vascular healing following intravascular lithotripsy. JACC Cardiovasc Interv.

